# Alterations in function and distribution of regulatory T Cells (Tregs) may blunt vaccine induced immune responses in HIV infection

**DOI:** 10.1186/1742-4690-9-S2-O43

**Published:** 2012-09-13

**Authors:** J van Lunzen, I Toth, P Hartjen, J Schulze zur Wiesch

**Affiliations:** 1 University Medical Center Hamburg-Eppendorf, Infectious Diseases Unit, Hamburg, Germany

## Background

Conflicting data exist about the frequency and role of regulatory T cells (Tregs) during the course of HIV infection. Tregs may substantially contribute to T cell homeostasis and fine regulation of T cell mediated immune responses.

## Methods

Peripheral blood and individual lymph nodes of a large cohort of HIV+ patients (n=131) at different disease stages, including 15 long-term nonprogressors and 21 elite controllers , was analyzed to determine the frequency, phenotype and function of Tregs.

## Results

A significantly increased relative frequency of Tregs within the CD4+ compartment of HIV+ patients in comparison with healthy controls (p<0.0001) was observed. The relative frequency of Tregs directly correlated with HIV viral load and inversely with CD4+ counts and was higher in corresponding lymph nodes. However, absolute Treg number was reduced in HIV infected patients vs. healthy controls (p<0.0001), but not in elite controllers (p>0.05). Loss of absolute Treg numbers was associated with increased markers of immune activation (HLA-DR, CD38 Ki-67)(p<0.0006). Initiation of antiviral therapy significantly increased absolute Treg numbers (p<0.0031). Moreover, we find that the expression of CD39 and CD73, newly defined ectonucleotidases involved in ATP degradation, correlated with progressive HIV disease, VL and immune activation. Of note, the capacity to suppress T cell proliferation in vitro was limited to the CD4+CD25highCD39+ T cell subset. Depletion of this distinct Treg subset in vitro resulted in a restoration of HIV specific T cell responses. Tregs of elite controllers exhibited the highest expression of CCR5, CTLA-4 and ICOS and the lowest level of CD39 indicating the functional importance of this ATP modulating enzymatic reaction.

## Conclusion

These data reconcile the seemingly contradictory results of previous studies on Tregs in HIV and highlight the complexity of Treg mediated immunoregulation. Blocking of ATP modulating molecules which are highly expressed on Tregs may restore HIV specific immune responses.

